# A giant ovarian cyst torsion: Case report

**DOI:** 10.1097/MD.0000000000033283

**Published:** 2024-04-12

**Authors:** He Hongju

**Affiliations:** a Department of Obstetrics & Gynecology, The Second Affiliated Hospital of Chongqing Medical University, Chongqing, China.

**Keywords:** adnexal torsion, case report, emergency surgery, gynecological acute abdomen, ovarian cyst torsion

## Abstract

**Introduction:**

Adnexal torsion (AT) is one of a gynecological condition characterized by an acute abdomen. Clinically, a giant ovarian cyst torsion with a diameter of 30 cm is rare. Therefore, an accurate and timely diagnosis and treatment are important.

**Patient concerns:**

A 25-year-old unmarried female, presented to the emergency department with intermittent abdominal cramps after a sudden change in position. Considering her symptoms and examination, ultrasound, and magnetic resonance imaging (MRI) results, ovarian cyst torsion was suspected.

**Diagnosis:**

Giant ovarian cyst torsion.

**Interventions:**

Surgical intervention with exploratory laparotomy was performed immediately.

**Outcomes:**

Intraoperatively, we found a 30-cm left ovarian cyst with a clear root. The left fallopian tube, infundibulopelvic ligament, and ovarian ligament were twisted 900 degrees. Finally, the pathological report revealed mucinous cystadenoma.

**Conclusion:**

Giant ovarian cyst torsion with a diameter of 30 cm is rare. Considering her symptoms and examination, ultrasound, and MRI results, ovarian cyst torsion was suspected. The patient was successfully treated using emergency surgery.

## 1. Introduction

Ovarian cyst torsion is an acute gynecological abdominal condition with an incidence of approximately 10%.^[1]^ It is prone to occur when the tumor pedicle is long, the tumor mobility is good, and the center of gravity is biased to 1 side. The typical symptom is sudden severe pain on 1 side of the lower abdomen after a change in position. Gynecological examination revealed a painful mass in the appendix. Many episodes of similar abdominal pain have been previously reported. Patients with adnexal torsion (AT) have different clinical manifestations owing to different tumor characteristics and degrees of blood flow obstruction, which can easily lead to misdiagnosis and missed diagnosis. Accurate and timely diagnosis and surgical treatment can prevent irreversible damage and destruction of ovarian function, which is especially important in patients with fertility requirements.

In this study, a 25-year-old unmarried female, presented to the emergency department with intermittent abdominal cramps after a sudden change in position. By combining her symptoms, examination, ultrasound, and magnetic resonance imaging (MRI) results, ovarian cyst torsion was considered. Emergency surgery was performed. Clinically, it is rare for ovarian cysts with a diameter of 30 cm to twist. Of course there is another possibility that the blood vessels are compressed, that the small blood vessels in the cyst may rupture and cause intratumoral hemorrhage, and that the tumor volume increases rapidly.

## 2. Case presentation

A 25-year-old unmarried female (gravida 0, para 0) presented to the emergency department with intermittent abdominal cramping after a sudden change in position. It has also been associated with vomiting. Last year, she had this similar symptoms 3 times; however, they were always mild and could remit after resting. Therefore, the patient did not receive any further therapy. She is currently menstruating. Her medical history included controlled hyperuricemia with etoricoxib. The patient had no significant family history.

**Figure 1.**
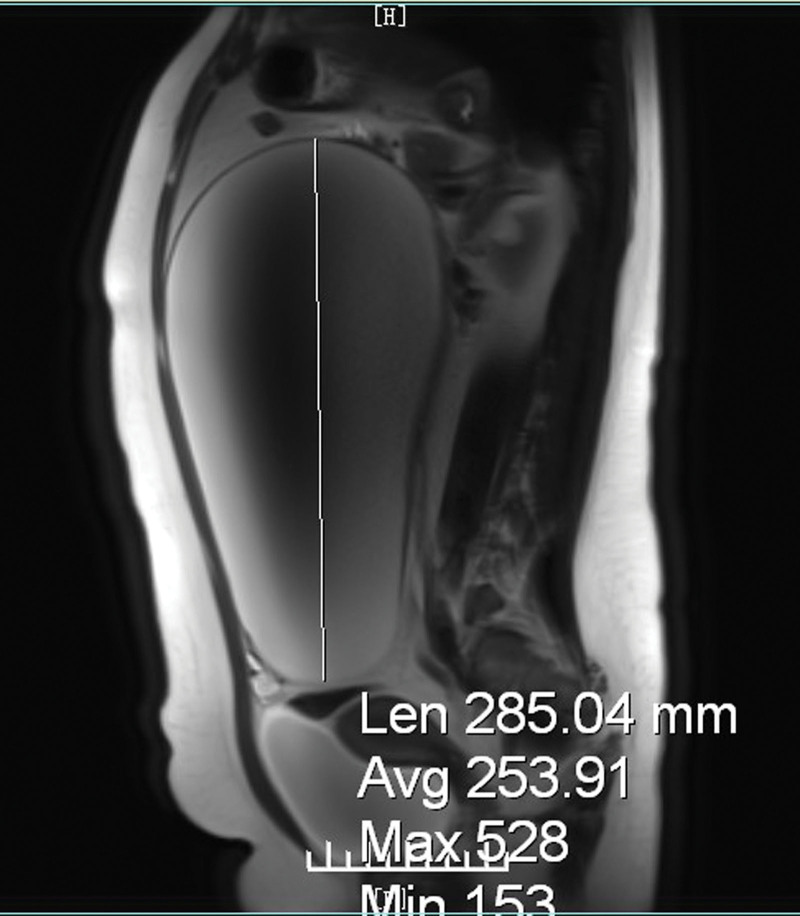
Magnetic resonance imaging of the abdomen and pelvis revealed a huge cystic mass in the adnexal torsion and a suspected cystadenoma.

The patient was hemodynamically stable and apyrexial. Examination revealed a tense grossly distended abdomen with left lower quadrant, without peritonism. Cardiorespiratory examination results were unremarkable. Laboratory tests showed a hemoglobin of 140 g/L, white blood cell count of 15.15 × 10^9^/L, neutrophil percentage of 75.8%, C-reactive protein of 78.71 mg/L, cancer antigen-125 of 36.5 U/mL and carcinoembryonic antigen of 0.46 μg/L. Urine HCG was negative. Amylase, lipase, and liver and renal function were normal. Ultrasound revealed a large cystic mass measuring 283 × 274 × 117 mm, and it had internal separation, and a blood vessel at its bottom. MRI of the abdomen and pelvis revealed a large cystic mass and a suspected cystadenoma (Fig. 1). Moreover, there was no free fluid in the abdominopelvic cavity, and no evidence of metastatic disease or dilated bowel loops. Considering the sudden abdominal cramping after changing position, a large adnexal cyst, low tumor markers and MRI report, a diagnosis of ovarian cyst torsion was made. Therefore, surgical intervention with exploratory laparotomy was immediately scheduled.

**Figure 2.**
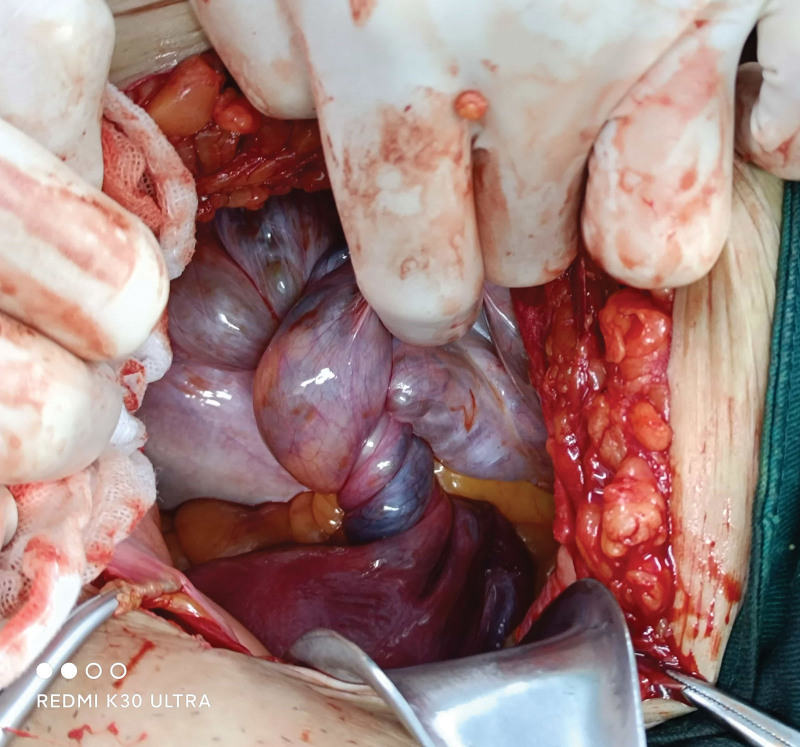
An intraoperative examination revealed torsion of the left ovary. The fallopian tube, infundibulo-pelvic ligament, and ovarian ligament are loosely twisted at 900°.

**Figure 3.**
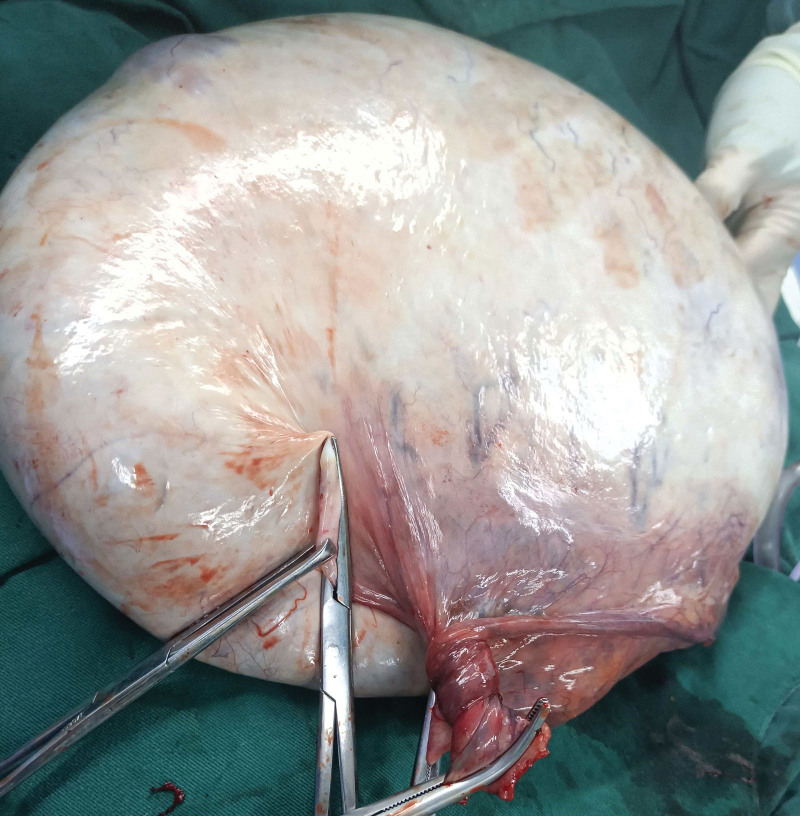
After removing fluid from the large cyst, the left ovary and salpinx were completely removed without resection or dissection.

Intraoperatively, we found a 30-cm left ovarian cyst with a clear root, that which showed was obviously congestion and edema. The ovarian surface was white. The left fallopian tube, infundibulo-pelvic ligament, and ovarian ligament loosely twist at 900° (Fig. 2). And pulsating feeling can be touched in the middle of torsion. Part of the dark brown fluid (2800 mL) was removed from the large cyst using a needle without splashing. The left ovary and salpinx were entirely removed without resection or dissection (Fig. 3). Finally, the pathological report revealed mucinous cystadenoma. The patient recovered uneventfully and was discharged 72h after surgery. Transvaginal ultrasonography was performed 30 days postoperatively, with no abnormal findings.

## 3. Discussion

AT tends to occur when the tumor has a long tip, good mobility, and the center of gravity is on 1 side, for example, in mature teratomas.^[2]^ Second, clinical ovarian cyst torsion is common in small cysts where there is enough space for torsion in the pelvic cavity, or when the cyst increases in size and enters the abdominal cavity from the pelvis, the space for activity increases and the sudden change of position leads to its torsion.^[3]^ The patient suddenly presented with severe left abdominal pain, unbearable pain with vomiting, and a large cyst. Therefore, an ovarian cyst was formed. However, torsion of such a huge cyst is rare. The postoperative autopsy specimen in this case showed that the cyst fluid was dark brown, which may have caused intra-tumor bleeding due to rupture of small blood vessels in the cyst and rapid increase in tumor volume.^[4]^

In practice, patients with ovarian cystic torsion have different clinical presentations due to different tumor characteristics and degrees of blood flow obstruction, which can easily lead to misdiagnosis and missed diagnosis. To improve the accuracy of ovarian cysts with tip torsion, Pierre P et al proposed a self-assessment questionnaire dedicated to gynecologic emergencies (SAQ-GE), which includes 5 independent predictors of AT diagnosis, which was used to assign a score of 3 for unilateral abdominal or low back pain, 2 for absence of abnormal leukorrhea and uterine bleeding, 2 for pain in the adnexal region, 2 for intolerable pain, and 1 for vomiting, for a total of 10 points for the diagnosis of AT.^[5]^ The risk of AT was 0.3% for SAQ-GE scores ≤ 6, 12.4% for SAQ-GE scores 7 to 9, and 52.2% for SAQ-GE scores of 10 in the high-risk group.^[5]^ Clinically, it is rare to encounter an ovarian cyst with a 30-cm and torsion in this case. In clinical practice, physicians should consider the diagnosis in conjunction with the patient symptoms, signs and examinations, and should not exclude the diagnosis of ovarian cyst torsion because of the large size of the ovarian cyst, thus missing the time for diagnosis and treatment, which may affect the fertility and ovarian function of women.

For AT, preservation of fertility and ovarian function in women is advocated, and for cases with mild torsion and a normal or light purple ovarian appearance, an increasing number of specialists choose to perform ovarian cyst nephrectomy.^[1]^ It has been reported that the incidence of pulmonary embolism is only 0.2% and that torsional repositioning does not increase the incidence of thromboembolic complications.^[6]^ The purple-black color seen with the naked eye does not indicate complete ischemic necrosis of the ovarian tissue, and 9l% to 100% of the ovarian tissue can be restored to function postoperatively after preservation.^[7]^ However, in this case, because the patient tumor was large, the ovary on the affected side was overly distended, and the normal ovarian tissue was severely damaged. Second, combined with the imaging findings and tumor markers of this patient, the possibility of mucinous cystadenoma of the ovary was considered to be high, and ovarian malignancy could not be completely excluded, so adnexal resection on the affected side was chosen. If the patient chooses to keep the affected ovary and perform detorsion with cystectomy, the affected ovary may not have normal function after surgery. If the pathological examination confirms mucinous cystadenoma of the ovary, there is a possibility of recurrence; if it is a malignant tumor, the patient needs to operate again. Despite the above considerations, despite the patient fertility requirements, she underwent salpingo-oophorectomy on the affected side after communicating with the patient and her family.

## Author contributions

**Conceptualization:** He Hongju.

**Investigation:** He Hongju.

**Project administration:** He Hongju.

**Resources:** He Hongju.

**Validation:** He Hongju.

**Writing – original draft:** He Hongju.

**Writing – review & editing:** He Hongju.
